# Metabolomics of the Protective Effect of *Ampelopsis grossedentata* and Its Major Active Compound Dihydromyricetin on the Liver of High-Fat Diet Hamster

**DOI:** 10.1155/2020/3472578

**Published:** 2020-01-28

**Authors:** Lanlan Fan, Xiaosheng Qu, Tao Yi, Yong Peng, Manjing Jiang, Jianhua Miao, Peigen Xiao

**Affiliations:** ^1^School of Pharmacy, Guangxi University of Chinese Medicine, Nanning 530001, China; ^2^National Engineering Laboratory of Southwest Endangered Medicinal Resources Development, Guangxi Botanical Garden of Medicinal Plants, Nanning 530023, China; ^3^School of Chinese Medicine, Hong Kong Baptist University, Kowloon Tong, Hong Kong, China; ^4^Institute of Medicinal Plant Development, Chinese Academy of Medical Sciences and Peking Union Medical College, 151 Malianwa North Road, Beijing 100193, China

## Abstract

The flavonoid dihydromyricetin (DMY) is the main component of *Ampelopsis grossedentata* (Hand-Mazz) W. T. Wang (AG), a daily beverage and folk medicine used in Southern China to treat jaundice hepatitis, cold fever, and sore throat. Recently, DMY and AG were shown to have a beneficial effect on lipid metabolism disorder. However, the mechanisms of how DMY and AG protect the liver during lipid metabolism disorder remain unclear. In this study, we first analyzed the chemical compounds of AG by HPLC-DAD-ESI-IT-TOF-MS^*n*^. Of the 31 compounds detected, 29 were identified based on previous results. Then, the effects of DMY and AG on high-fat diet hamster livers were studied and the metabolite levels and metabolic pathway activity of the liver were explored by ^1^H NMR metabolomics. Compared to the high-fat diet group, supplementation of AG and DMY attenuated the high-fat-induced increase in body weight, liver lipid deposition, serum triglycerides and total cholesterol levels, and normalized endogenous metabolite concentrations. PCA and PLS-DA score plots demonstrated that while the metabolic profiles of hamsters fed a high-fat diet supplemented with DMY or AG were both far from those of hamsters fed a normal diet or a high-fat diet alone, they were similar to each other. Our data suggest that the underlying mechanism of the protective effect of DMY and AG might be related to an attenuation of the deleterious effect of high-fat diet-induced hyperlipidemia on multiple metabolic pathways including amino acid metabolism, ketone body metabolism, energy metabolism, tricarboxylic acid cycle, and enhanced fatty acid oxidation.

## 1. Introduction

*Ampelopsis grossedentata* (Hand-Mazz.) W. T. Wang (AG), also known as Vine tea, is a plant species mainly distributed in central and southern China which is rich in flavonoids, polysaccharides, and polyphenols. First used by the Zhuang and Yao people [1], it became later also widely used in Tujia, Lahu, Dong, Jinuo, and Hakka areas. Besides consumption as a tea prepared from the tender stems and leaves, AG is also used to treat jaundice hepatitis, cold fever, and sore throat. Next too its antioxidant [2] and anti-inflammatory effects [3], it can also normalize blood lipid [4] and sugar [5] levels and attenuate liver injuries [6].

Dihydromyricetin (DMY), which is the main flavonoid of AG with a content of up to 35% [7], showed antiproliferation capacities in lung [8], breast, [9], and ovarian cancer [10], improvements on hypertension [11], hyperlipidemia [12, 13] and abnormal blood sugar levels [14], as well as neuroprotective activity against Alzheimer's disease [15], Parkinson's disease [16], alcohol addiction, depression [17], and dermatoprotection [18]. In addition, a preventive effect on myocardial fibrosis [19], myocardial hypertrophy [20], and cardiac ischemia/reperfusion injury [21] was reported.

The liver plays an important role in the digestion, absorption, oxidation, decomposition, and transformation of nutrients such as lipids, proteins, and sugars [22]. Abnormal accumulation and distribution of lipids and their metabolites in the body result in lipid metabolic disorder which is an important inducer of diabetes, atherosclerosis, nephrotic syndrome, and cardio- and cerebrovascular disease. Considering the health impact of chronic metabolic diseases such as hyperlipidemia, safe and reliable functional compounds from natural sources are highly needed. Based on the remarkable blood lipid regulating characteristics described in the literature [23–26], both AG and DMY might possess great health-promoting potentials; however, their capacity to prevent liver injury as well as the underlying mechanisms is unknown.

Metabolomics, reflecting the overall changes of the organism by studying the changes of endogenous metabolite levels, has recently played an important role in revealing the pharmacodynamics and mechanism of some disease [27–29] or traditional Chinese medicine [30].

Since up to 85% cholesterol is synthesized extrahepatic in male hamsters, hyperlipidemia can be caused in a relatively short period of time. Thus, as the lipid metabolism of hamsters is similar to that of human, especially male hamsters are widely used as a model organism of lipid metabolism disorder [31, 32].

In this study, the chemical compounds of AG were first analyzed by liquid chromatography-mass spectrometry. Then, the lipid metabolism disorder model of hamster was induced by high-fat diet, and the preventive effect of AG and DMY on hyperlipidemia and hyperglycemia was studied by liver metabolomics.

## 2. Materials and Methods

### 2.1. Chemicals and Materials

Acetonitrile and formic acid, both LC-MS grade, were purchased from Fisher (Fair Lawn, New Jersey, USA) and Sigma-Aldrich (St. Louis, USA), respectively. For sample extraction, analytical grade methanol and ethanol was used while deionized water was obtained from a Milli-Q water purification system (Millipore, Bedford, MA, USA).

**Figure 1:**
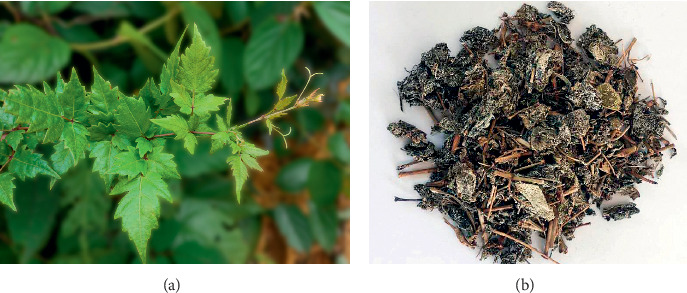
Photograph of fresh (a) and dried (b) *Ampelopsis grossedentata*.

*Ampelopsis grossedentata* (Hand-Mazz.) W. T. Wang (AG) was purchased from Dayaoshan natural plant development Co. Ltd. (Guangxi, China) and identified by Professor Yi Cai from Guangxi University of Chinese Medicine (Figure 1). Dihydromyricetin (DMY) was purchased from Chengdu preferred Biological Technology Co., Ltd. (Chengdu, China) with purity above 98%.

### 2.2. Sample Preparation

For LC-MS analysis, the AG sample was crushed through an 80-mesh sieve and dried at 50°C to constant mass. Next, 0.5 g powder was dissolved in 50 mL of methanol and ultrasonically extracted for 40 min. The extract was filtered, the primary filtrate was discarded, and the filtrate was diluted 100 times and filtered through a 0.22 *μ*m filter. For the animal experiments, 150 g AG power was soaked in 5000 mL boiling water for 15 min and concentrated to 0.6 g crude drug/ml.

### 2.3. HPLC-DAD-ESI-IT-TOF-MS^*n*^ Analysis

High-performance liquid chromatography with a diode array detector and combined with electrospray ionization ion trap time-of-flight multistage mass spectrometry (HPLC–DAD–ESI-IT-TOF-MS^*n*^) analyses was performed with a Shimadzu LCMS-IT-TOF instrument including two LC-20AD pumps, a SIL-20AC autosampler, a CTO-20A column oven, a SPD-M20A PDA detector, a CBM-20A system controller, an ESI ion source, and an IT-TOF mass spectrometer (Shimadzu, Kyoto, Japan).

The chromatography separations were performed on a Merck Purospher STAR RP-18 column (250 mm × 4.6 mm, 5 *μ*m) with a column temperature of 40°C. The mobile phase consisted of 0.1% formic acid (v/v) (A) and acetonitrile (B) using a gradient program of 50–58% B in 0–17 min, 58–70% B in 17–20 min, 70–85% B in 20–26 min, and 85–90% B in 26–35 min. The solvent flow rate was 1.0 mL/min. The PDA detector wavelength was 350 nm.

For ESI-IT-TOF-MS^*n*^ analysis, the mass spectrometer was programmed to execute a full scan over *m/z* 100–1000 (MS^1^) and *m/z* 50–1000 (MS^2^ and MS^3^) in both positive-ion (PI) and negative-ion (NI) detection modes with the following settings: a flow rate of 0.2000 mL/min, a heat block and curved desolvation line temperature of 250°C, a flow rate of the nebulizing nitrogen gas of 1.5 L/min, an interface voltage of (+) 4.5 kV and (−) −3.5 kV, a detector voltage of 1.70 kV, an ion accumulation time of 20 ms, and a relative collision-induced dissociation energy of 50%. Trifluoroacetic acid sodium solution (2.5 mM) was used to calibrate the mass range from 50 to 1000 Da. All data were recorded and analyzed with Shimadzu LCMS solution Version 3.60, Formula Predictor Version 1.2, and Accurate Mass Calculator (Shimadzu, Kyoto, Japan).

After collecting LC/MS data, the mass spectrum of each chromatographic peak was extracted based on excimer ions ([M + H]+, [M − H]−) and loading ions ([M + NH_4_]^+^, [M + Na]^+^, [M + Cl]^−^, [M + HCOO]^−^, etc.). With PeakView 1.2 software, the relative molecular mass of the primary mass spectrometry was obtained and the molecular formula was fitted in the mass deviation range of 5 × 10^−6^. The chemical compounds of AG and its genus were then collected and sorted out with the Scifinder and Reaxys databases. Next, secondary mass spectrometry information of the chromatographic peak and the corresponding fragment ions of the compound were obtained and the chemical composition was compared with the literature based on the cleavage of the ions and the reference substance.

### 2.4. Animal Experiments

Animal care and procedures were approved by and conducted according to the standards of the Guangxi University of Chinese Medicine (Nanning, China). Male LVG hamsters (110–130 g, 8 weeks old, Vital River Laboratory Animal Technology Co., Ltd., Beijing, China) were maintained in a temperature-controlled (22–25°C) room on a 12 h : 12 h light-dark cycle with food and water ad libitum. One week after adaptive feeding, the hamsters were randomly divided into five groups with six animals in each group: normal diet group (ND), high-fat diet group (HFD), high-fat diet supplemented with *Ampelopsis grossedentata* group (2 g/kg.d) (HFD + AG), high-fat diet supplemented with dihydromyricetin group (173 mg/kg.d) (HFD + DMY), and high-fat diet supplemented with simvastatin group (2.5 mg/kg.d) (HFD + ST). The AG dose was derived through dose conversion of literature data [1], whereas the DMY dose was calculated based on its content in AG. The high-fat diet consisted of 73.5% basic feed, 24.5% lard, and 2% cholesterol. At week 8, the animals were fasted for 12 hours and anesthetized with intraperitoneally injection of 3% pentobarbital sodium (0.3 mL/100 g), and blood was drawn from the ophthalmic plexus veins. The blood samples were centrifuged at 3000 rpm for 10 min, and the serum levels of total cholesterol (TC), triglycerides (TG), high-density lipoprotein (HDL), low-density lipoprotein (LDL), aspartate aminotransferase (AST), and alanine aminotransferase (ALT) were measured by the Automatic Biochemical analyzer (HITACHI 7600, Hitachi High-Tech Co., Ltd., Japan). After the blood was collected, the animals were sacrificed and the liver of each group was weighed, frozen sections were taken, and oil red O staining was used to mark the fat. The remaining samples were frozen in liquid nitrogen and stored at −80°C. Hepatic lipids including hepatic TC and TG were measured by commercial kits (Applygen Technologies Inc., Beijing, China).

### 2.5. Liver Metabolomics

Fifty milligrams frozen dried liver sample powder was washed twice in 1000 *μ*L purified water and centrifuged at 13000 rpm for 15 min. After the last wash, 450 *μ*L supernatant was mixed with 50 *μ*L DSS (4,4-dimethyl-4-silapentane-1-sulfonic acid) standard solution (Anachro, Canada). All NMR spectra were acquired using a standard Bruker noesygppr1d pulse sequence on a Bruker AV III 600 MHz spectrometer equipped with an inverse cryoprobe operating at 600.13 MHz (Bruker Biospin, Milton, Canada). In total, 256 scans were collected into 32768 data points over a spectral width of 8000 Hz. Fourier transformation, phase adjustment, and baseline correction of the ^1^H NMR free induction decay (FID) signal was done with Chenomx NMR suit (version 8.1, Chenomx, Edmonton, Canada). DSS-d6 peak (0.0 ppm) was used as the standard for all chemical shifts of the spectrograms. Reverse convolution was performed to adjust the peak shape. The variable matrix was used as the source data for subsequent PCA and PLS-DA analysis. The distribution and relative quantification of the identified metabolites were analyzed by Ward's Hierarchical Clustering and visualized with a heatmap. In the heatmap, the red and blue colors indicate higher and lower relative content compared to the average value, respectively. The identification of biomarkers and the analysis of metabolic pathways were carried out using the Human Metabolomics Database (HMDB) and the Kyoto Encyclopedia of Genes and Genomes (KEGG). The metabolic pathways were visualized using MetPA network software (http://metpa.metabolomics.ca/) [33]. Each circle in the graph represents a differential metabolic pathway obtained through metabolic pathway analysis, and the color of each circle represents the magnitude of *p* value: the more red the color, the smaller the *p* value (see supplementary material). The higher the significance, the larger the value of the corresponding −log(*p*). The size of the circle indicates the influence of the metabolic pathway, thus the bigger the circle, the greater its influence. As such, the closer the position of the circle is to the upper right side of the diagonal line in the resulting figure, the more likely the metabolic pathway is biologically relevant.

### 2.6. Statistical Analysis

The data were expressed as the mean ± standard error of the mean (SEM). One-way ANOVA was used to analyze significant differences among multiple groups, while couple comparisons were performed via the *t* test. Statistical values of *p* < 0.05 and *p* < 0.01 were considered significant.

## 3. Results

### 3.1. HPLC-DAD-ESI-IT-TOF-MS^*n*^ Analysis of *Ampelopsis grossedentata*

**Figure 2:**
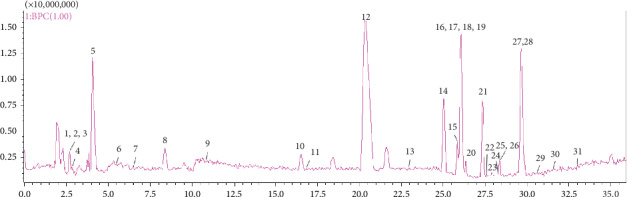
BPI chromatograph of *Ampelopsis grossedentata*.

**Figure 3:**
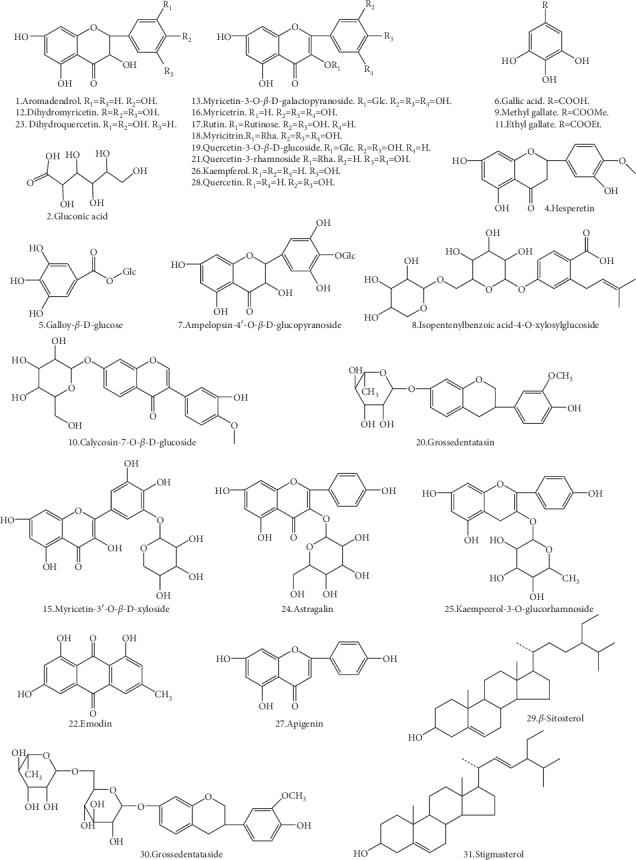
Structures of dihydromyricetin and identified compounds isolated from *Ampelopsis grossedentata*.

For the detection of the chemical compounds of AG, both the positive- and negative-ion modes of the mass spectrometer were tested. The AG sample was analyzed under the “2.3” chromatographic and mass spectrometric conditions to obtain the base peak intensity (BPI) chromatograms in HPLC-DAD-ESI-IT-TOF-MS^*n*^ (Figure 2.) In total, 31 flavonoid compounds were obtained of which 29 could be identified based on our previous results [34] and literature data [35–40] (Table 1 and Figure 3). Of note, the retention time of DMY was 20.387 min.

### 3.2. Analysis of AG and DMY on Physiology of Hamsters on a High-Fat Diet

LVG hamsters were randomly divided into five groups with six animals in each group: a normal diet group (ND), a high-fat diet group (HFD), a high-fat diet group supplemented with 2 g/kg.d AG (HFD + AG), a high-fat diet group supplemented with 173 mg/kg.d DMY (HFD + DMY), and a high-fat diet group supplemented with 2.5 mg/kg.d simvastatin (HFD + SV). The water extract of AG and DMY was given from the start of the high-fat diet to check their preventive effects, while SV was given two weeks after the lipid metabolism disorder model was set up.

The obtained ^1^H NMR sample spectra were analyzed in combination with the Chenomx self-contained database. In total, 66 metabolites and their corresponding absolute concentration values were obtained in liver samples (Supplementary material Figures 1 and 2).

**Figure 4:**
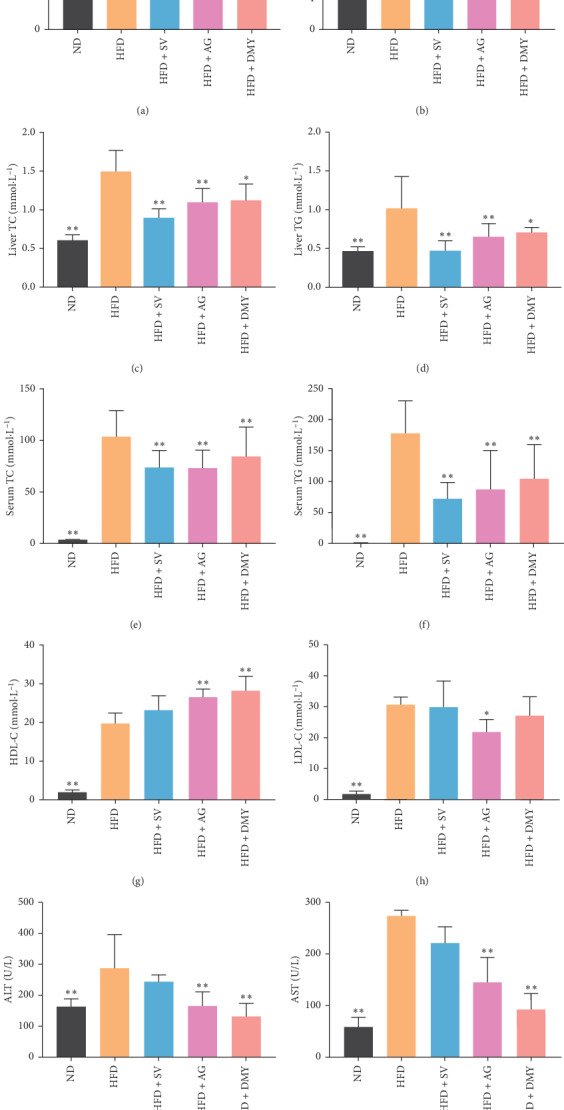
Effect of high-fat diet and high-fat diet in combination with AG, DMY, or SV on body weight (a), liver weight (b), liver TC (c), liver TG (d), serum TC (e), serum TG (f), HDL (g), LDL (h), ALT (i) and AST (j). Data are presented as the mean ± SEM. ^*∗*^*p* < 0.05, ^*∗∗*^*p* < 0.01 compared to HFD, *n* = 6.

Earlier, a dyslipidemia hamster model was produced after 2 weeks of high-fat diet [31]. After 8 weeks, at the end of the experiment, the blood serum of the animals fed a high-fat diet was lipemic, showing a milky white semisolid (data not shown). Compared to ND, a significant increase in the body weight, liver index, liver TC and TG, serum TC, TG, HDL, LDL, ALT, and AST levels was observed in HFD, HFD + AG, HFD + DMY, and HFD + SV (Figure 4). Compared to HFD, body weight, liver index, liver TC and TG, serum TC, TG, LDL, ALT, and AST levels were significantly lower in HFD + AG and HFD + DMY. Compared to HFD, HDL was higher in HFD + AG and HFD + DMY (Figure 4). Although most effects of AG and DMY on regulating blood lipids and hepatic lipids were similar to those observed with SV, AG and DMY had more beneficial effects on HDL, AST, and ALT than SV.

During anatomy, the shape and color of the liver of the individual animals were examined. Livers of ND showed a glossy dark red color and had a greasy feeling, a regular shape, sharp edges, and a grainy surface. Livers of HFD were larger compared to those of ND and had a yellow-brownish color, and the tissue cut surface had a greasy feeling. Livers of HFD + AG and HFD + DMY were larger compared to those of ND and had a pink color and a regular shape. The livers of HFD + SV were smaller compared to those of HFD and had a yellow-brownish color and granular bumps on the surface.

**Figure 5:**
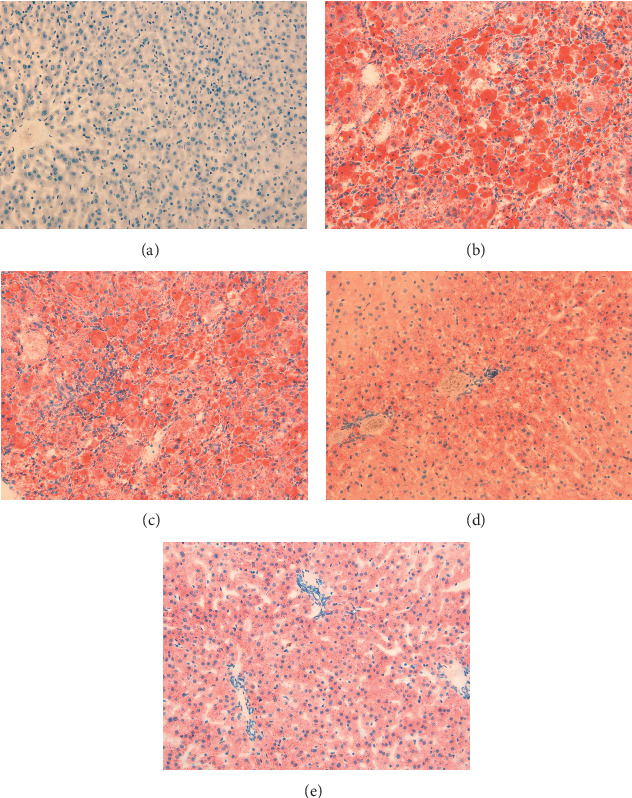
Oil red O staining of liver pathology sections indicated that AG and DMY improved hepatic lipid profiles. (a) ND; (b) HFD; (c) HFD + SV; (d) HFD + AG; (e) HFD + DMY. Oil red O staining results in visualization of neutral fat in red.

Oil red O staining indicated that livers from HFD had more lipid deposition compared to ND (Figures 5(a) and 5(b)). Livers from HFD + AG and HFD + DMY had less lipid deposition compared to HFD (Figures 5(c) and 5(d)). Livers from HFD + SV had less lipid deposition compared to HFD, although larger fat particles were still visible (Figure 5(e)).

### 3.3. Analysis of AG and DMY on Liver Metabolome of Hamsters on a High-Fat Diet

**Figure 6:**
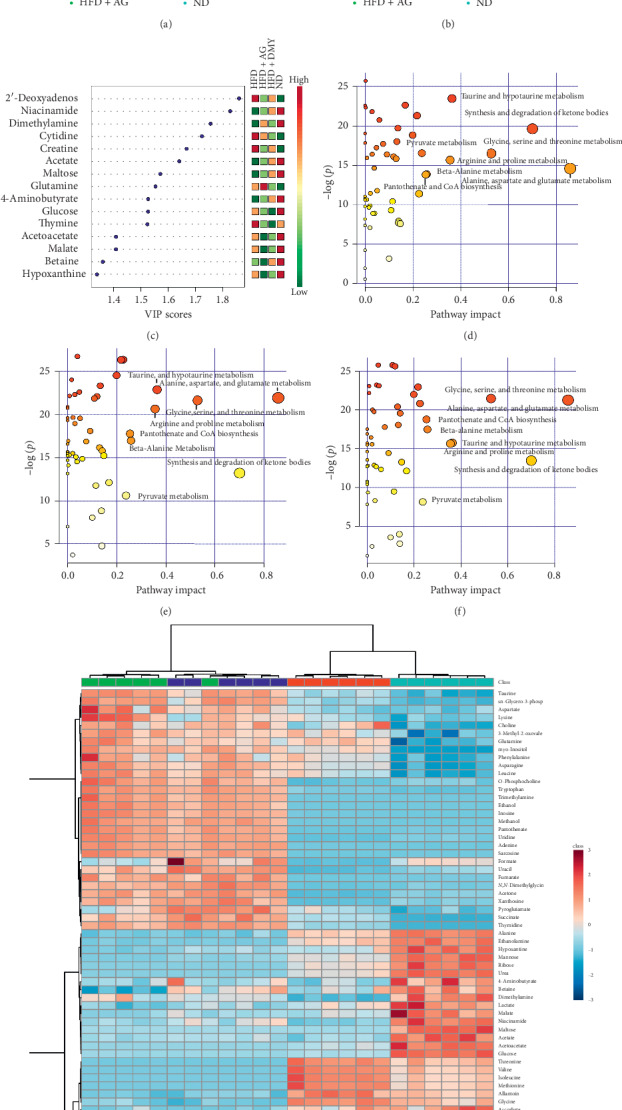
^1^H NMR metabolism analysis of the hamster liver. (a) PCA score (ND vs HFD vs HFD + AG vs HFD + DMY); (b) PLS-DA score (ND vs HFD vs HFD + AG vs HFD + DMY); (c) PLS VIP (ND vs HFD vs HFD + AG vs HFD + DMY); (d) pathway analysis (ND vs HFD); (e) pathway analysis (HFD vs HFD + AG); (f) pathway analysis (HFD vs HFD + DMY); (g) heatmap analysis (ND vs HFD vs HFD + AG vs HFD + DMY).

Endogenous metabolites in the liver of hamsters belonging to the different experimental groups were detected by ^1^H NMR (Table 2). Unsupervised PCA and PLS-DA, used for dimensionality reduction of multidimensional data, indicated that ND and HFD were separated from each other suggesting that the generation of the high-fat diet model was successful (Figures 6(a), and 6(b)). In addition, HFD + AG and HFD + DMY were separated from HFD, indicating that the lipid metabolism disorder of the high-fat diet hamster was partially alleviated or contained through AG or DMY supplementation (Figures 6(a), and 6(b)). There was little overlap between the 95% confidence region of HFD + AG and HFD + DMY, indicating that both groups had similar effects on liver metabolism of animals on high-fat diet.

Based on the results of the cluster analysis, the distribution and relative quantification of the identified metabolites were visualized with a heatmap. Similar to PCA and PLS-DA, ND and HFD belonged to two different clusters in the heatmap, while there was some overlap between HFD + AG and HFD + DMY (Figure 6(g)).

Variable importance in projection (VIP) is a weighted sum of squares of the PLS loadings taking into account the amount of explained Y-variation in each dimension and shows important features identified by PLS-DA (Figure 6(c)). After treatment with high-fat diet, the affected metabolic pathways included synthesis and degradation of ketone bodies, alanine, aspartate and glutamate metabolism, glycine, serine, and threonine metabolism, taurine and hypotaurine metabolism, D-glutamine and D-glutamate metabolism, arginine and proline metabolism, beta-alanine metabolism, pantothenate and CoA biosynthesis, and pyruvate metabolism (Figure 6(d), details are shown in Supplementary material Table 1).

**Figure 7:**
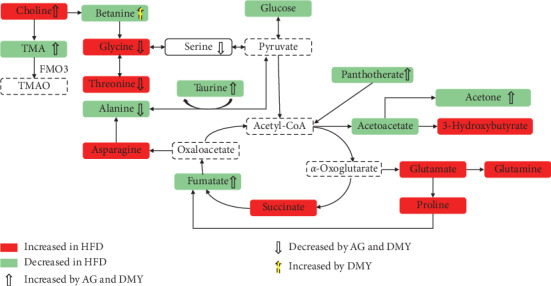
Summary of the findings of the metabolomic pathway analysis. See text for details.

Comparing HFD to HFD + AG or HFD + DMY, respectively, indicated that the effects of the different treatments on high-fat diet were overlapping. Affected metabolic pathways included alanine, aspartate, and glutamate metabolism; synthesis and degradation of ketone bodies; glycine, serine, and threonine metabolism; taurine and hypotaurine metabolism; arginine and proline metabolism; *β*-alanine metabolism; pantothenate and CoA biosynthesis; and pyruvate metabolism (Figure 6(e) and 6(f), Supplementary material Tables 2 and 3). Interestingly, supplementation of high-fat diet with AG or DMY resulted in a huge increase of trimethylamine (TMA) (Figure 7 and Table 2) compared to HFD.

## 4. Discussion

The current study consisted of two parts. First, the chemical composition of AG was analyzed by HPLC-DAD-ESI-IT-TOF-MS^*n*^. Then, the effect of AG and DMY on the metabolome of livers from high-fat diet hamsters was analyzed by ^1^H NMR.

### 4.1. Analysis of the Chemical Composition of AG

With HPLC-DAD-ESI-IT-TOF-MS^*n*^, 31 compounds were detected in AG extracts, 29 of which could be identified based on our previous results [34] and literature [35–40]. Of these 29 identified compounds, most were flavonoids, in addition to a small amount of polyphenols, triterpenoids, and others. The presence of calycosin-7-*O*-*β*-*D*-glucoside (10^#^) in AG was reported for the first time in [41].

### 4.2. Effect of High-Fat Diet on the Liver of Hamster

In male hamsters, 85% of cholesterol derives from extrahepatic synthesis sources meaning that hyperlipidemia can be induced in a relatively short period of time. Moreover, the lipid metabolism of hamsters is similar to that of human suggesting male hamsters are a suitable model organism of lipid metabolism disorder [31, 32]. In the current study, lipid metabolism disorder was induced in male hamsters by feeding them a high-fat diet consisting of 24.5% lard and 2% cholesterol. Because of the high content of fat and cholesterol, the blood serum became lipemic at the end of the treatment period. Animals on a high-fat diet had increased body and liver weight, higher serum levels of TC and TG, and more lipid deposition compared to control suggesting that our lipid metabolism disorder model was successfully established.

The effect of high-fat diet on the metabolome of the liver was analyzed with ^1^H NMR. The results show that the content of glutamic acid and aspartic acid increased while that of alanine decreased (Figure 7 and Table 2).

As fat intake increases, free fatty acids formed by increased lipid hydrolysis undergo beta-oxidation to produce acetyl coenzyme A (CoA) which then condenses with oxaloacetic acid and further converts into alpha-ketoglutarate via tricarboxylic acid (TCA) cycling. Alpha-ketoglutarate can be aminated or transaminated to produce glutamic acid whereas oxaloacetic acid can be converted into aspartic acid.

Due to the high fat intake, the utilization of glucose in the animal is reduced and subsequently the synthesis of hepatic glycogen in the liver is increased. Consequently, this leads to a decreased glucose levels and, ultimately, alanine synthesized from pyruvate by alanine transaminase decreases.

The levels of the ketone bodies, especially 3-hydroxybutyric acid—intermediates produced in the liver during fatty acid oxidation—were higher in hamsters on high-fat diet (Figure 7). In the extrahepatic tissues, these ketone bodies undergo ketosis to form acetyl-CoA. If present in large amounts, acetyl-CoA inhibits the activity of the pyruvate dehydrogenase complex and restricts the utilization of sugar. At the same time, acetyl-CoA can activate pyruvate carboxylase and promote gluconeogenesis. Extrahepatic tissues use ketone body oxidation to supply energy, which on its turn reduces the demand for glucose.

However, conversion of fat into sugar is inefficient. In fact, due to the energy need to maintain normal physiological functions, depleted blood glucose levels during prolonged hyperlipidemia was shown before [35, 42].

Next to decreased glucose levels, the levels of succinic acid in the TCA cycle increased, whereas the levels of fumaric acid and maleic acid decreased (Figure 7).

Taken together, the results indicate that high-fat diet in hamsters results in a disturbed lipid metabolism, accompanied by a disturbed amino acid, sugar, and energy metabolism (Figure 7).

### 4.3. Compensatory Effect of AG and DMY on the Liver of Hamsters on a High-Fat Diet

In the present study, the effect of AG and DMY on high-fat diet-induced lipid metabolism disorder was analyzed. Compared to animals with high-fat diet only, supplementation of high-fat diet with AG or DMY resulted in lower body weight and liver index, lower levels of liver TC and TG, lower levels of serum TC, TG, LDL, ALT, and AST, increased levels of serum HDL, and less liver lipid depositions (Figures 4 and 5). Furthermore, a boost of the high-fat diet-induced increase in fatty acid oxidation and the synthesis of alpha-ketoglutarate, glutamic acid, and aspartic acid was observed in those animals supplemented with AG or DMY (Figure 7). Increased levels of alpha-ketoglutarate resulted in higher levels of succinic acid and fumaric acid in the TCA cycle. Co-administration of AG or DMY during high-fat diet prevented the increase of 3-hydroxybutyric acid, acetoacetate, and acetone slightly, suggesting reduced ketone body synthesis.

In addition, compared to animals fed a high-fat diet, supplementation of AG or DMY during high-fat diet resulted in lower levels of serine, glycine, and threonine (Figure 7). Since these amino acids can be converted to pyruvic acid and subsequently glycogen, it is suggested that supplementation of AG or DMY preserves liver glycogen production during high-fat diet.

Taurine can promote the oxidative decomposition of fatty acids by neutralizing cholic acid in the liver and leads to the formation of taurocholic acid. Taurocholic acid is then secreted into the digestive tract with bile to promote the digestion and absorption of fat and fat-soluble vitamins. Metabolism of pantothenic acid results in the formation of CoA. Considering the central role of CoA in fatty acid beta-oxidation, a lack of pantothenic acid inhibits lipid metabolism. In addition to the increased taurine levels induced by high-fat diet, which promote fat metabolism, supplementation of AG or DMY during high-fat diet resulted in a further increase of taurine levels compared to animals on a high-fat diet only (Figure 7). Moreover, while high-fat diet led to a decrease in pantothenic acid levels, supplementation of AG or DMY during high-fat diet could maintain the pantothenic acid levels (Figure 7). Combined, these data suggest that supplementation of AG or DMY during high-fat diet can significantly increase fatty acid oxidation.

Supplementation of high-fat diet with AG or DMY resulted in a huge increase of trimethylamine (TMA) (Table 2) compared to HFD. TMA is synthesized from dietary sources such as carnitine or choline by the microbiota. Interestingly, higher levels of choline were observed in liver of HFD + Ag or HFD + DMY compared to HFD (Table 2). Next, TMA is converted to trimethylamine *N*-oxide (TMAO) by hepatic flavin monooxygenases 3 (FMO3) [43]. In fact, TMAO was shown earlier to attenuate high-fat high-cholesterol diet-induced steatohepatitis through modulating the gut microbiota and as such reducing cholesterol absorption and hepatic cholesterol overload in rats [44]. Thus, our data might suggest that enhanced TMA levels could be another mechanism by which AG or DMY might be beneficial for lipid metabolic disorder.

Taken together, our data suggest that the protective effect of AG and DMY on the liver during high-fat diet might act by preserving the efficacy of multiple metabolic pathways including enhanced fatty acid oxidation and improved amino acid metabolism and ketone body synthesis.

## 5. Conclusions

In the current study, 31 chemicals were detected and 29 flavonoids were identified in *Ampelopsis grossedentata* by HPLC-DAD-ESI-IT-TOF-MS^*n*^. When supplemented to hamsters on a high-fat diet, *Ampelopsis grossedentata* and its main active ingredient dihydromyricetin could control the body weight and reduce the blood levels of TC and TG. Liver slice staining indicated that both *Ampelopsis grossedentata* and dihydromyricetin could effectively inhibit the formation of hepatic fat granules. Metabolomics studies suggested that *Ampelopsis grossedentata* and dihydromyricetin can influence the metabolism of hamsters. Our data indicated that the mechanism of *Ampelopsis grossedentata* and dihydromyricetin is basically overlapping and might involve the regulation of liver lipid metabolism during a high-fat diet by modulating amino acid metabolism, promoting fatty acid oxidation, promoting the increase of pantothenic acid and taurine, and enhanced levels of trimethylamine. Our results provide a scientific basis for elucidating the mechanism of action by which vine tea and DMY might be able to improve lipid metabolism disorders.

## Figures and Tables

**Table 1:** Characterization of compounds detected in *Ampelopsis grossedentata* extract by HPLC-DAD-ESI-IT-TOF-MS^*n*^.

No.	*t*_R_ (min)	Formula	PI pred. (Da)	PI meas. (Da)	Error (ppm)	Major fragment ions	Identification
1.	2.725	C_15_H_12_O_6_	289.0707	289.0711^a^	1.51	275.0607, 245.0654, 203.0556, 185.0416	Aromadendrol
2.	2.725	C_6_H_12_O_7_	195.0510	195.0508^b^	1.15	177.0417, 159.0328, 113.0227	Gluconic acid
3.	2.725	C_21_H_16_O_6_	365.1020	365.1002^a^	4.85	305.0793, 203.0556, 185.0416	—
4.	2.950	C_16_H_14_O_6_	301.0718	301.0744^b^	8.73	173.0267	Hesperetin
5.	4.230	C_13_H_16_O_10_	333.0816	333.0814^a^	0.67	169.0153, 125.0248	Galloy-*β*-D-glucose
6.	5.487	C_7_H_6_O_5_	169.0412	169.0148^b^	3.25	125.0288	Gallic acid
7.	6.490	C_21_H_22_O_13_	481.0988	481.0990^b^	0.49	463.0852, 319.0421, 301.0252, 193.0132	Ampelopsin-4′-*O*-*β*-*D*-glucopyranoside
8.	8.318	C_23_H_32_O_12_	499.1821	499.1792^b^	5.80	385.1851, 205.1231, 161.0500	Isopentenyl benzoic acid-4-*O*-xylosylglucoside
9.	10.770	C_8_H_8_O_5_	183.0299	183.0295^b^	2.16	183.0318	Methyl gallate
10.	16.545	C_22_H_22_O_10_	491.1195	491.1180^c^	3.36	283.0573, 268.0368, 239.0307	Calycosin-7-*O*-*β*-*D*-glucoside
11.	16.887	C_9_H_10_O_5_	197.0455	197.0411^b^	7.31	—	Ethyl gallate
12.	20.387	C_15_H_12_O_8_	319.0459	319.0443^b^	5.13	301.0335, 193.0140, 179.0056	Dihydromyricetin
13.	22.912	C_21_H_20_O_13_	481.0977	481.0956^a^	4.31	319.0408, 165.0210	Myricetin-3-*O*-*β*-*D*-galactopyranoside
14.	25.028	C_27_H_36_O_7_	471.2388	471.2383^b^	1.12	237.0389, 175.0377	—
15.	25.778	C_20_H_18_O_12_	449.0725	449.0711^b^	9.57	273.0467, 245.0405, 217.0501	Myricetin-3′-*O*-*β*-*D*-xyloside
16.	26.003	C_15_H_10_O_8_	319.0448	319.0429^a^	6.11	273.0398, 245.0447, 217.0471, 165.1365, 153.0194	Myricetin
17.	26.003	C_27_H_30_O_16_	611.1607	611.1664^a^	9.40	321.0481, 301.0311, 165.0195, 153.0194	Rutin
18.	26.062	C_21_H_20_O_12_	463.0882	463.0868^b^	3.02	463.0851, 317.0285, 287.0180, 271.0246	Myricitrin
19.	26.062	C_21_H_20_O_12_	463.0882	463.0868^b^	3.02	316.0261, 301.0307, 271.0246	Quercetin-3-*O*-*β-D-*glucoside
20.	26.287	C_22_H_26_O_8_	417.1555	417.1536^b^	4.52	318.0308, 273.0726, 262.0683	Grossedentatasin
21.	27.328	C_21_H_20_O_11_	447.0933	447.0940^b^	1.60	447.0878, 301.0307, 271.0264	Quercetin-3-rhamnoside
22.	27.553	C_15_H_10_O_5_	269.0455	269.0433^b^	8.32	241.0401, 225.0529	Emodin
23.	27.920	C_15_H_12_O_7_	303.0510	303.0490^a^	6.66	287.0542, 153.0206, 123.0509	Dihydroquercetin
24.	28.303	C_21_H_20_O_11_	447.0933	447.0963^a^	0.70	285.0380, 255.0276, 227.0326, 151.0052	Astragalin
25.	28.362	C_21_H_20_O_10_	433.1129	433.1097^a^	7.46	165.0204, 153.0155, 147.0379, 129.0772	Kaempeerol-3-*O*-glucorhamnoside
26.	28.362	C_15_H_10_O_6_	287.0550	287.0518^a^	11.24	241.0500, 213.0587, 165.0240, 153.0155, 121.0289	Kaempferol
27.	29.637	C_15_H_10_O_5_	269.0455	269.0433^b^	8.32	227.0373, 183.0453, 149.0235	Apigenin
28.	29.795	C_15_H_10_O_7_	301.0354	301.0334^b^	6.54	273.0354, 229.0490, 165.0176, 137.0275	Quercetin
29.	30.678	C_29_H_50_O	415.3934	415.3909^a^	6.14	367.2291, 317.2050171.1095	*β*-Sitosterol
30.	31.563	C_28_H_36_O_13_	579.2083	579.2124^b^	7.04	271.1662, 245.1892, 135.0843	Grossedentataside
31.	32.908	C_29_H_48_O	411.3632	411.3628^b^	1.07	393.2614, 368.3089, 213.1123	Stigmasterol

Note: ^a^[M + H]^＋^; ^b^[M − H]^−^; ^c^[M + COOH]^−^.

**Table 2:** Trends of metabolites concentration in liver of hamsters.

No.	Metabolites	HFD/ND	Trend	AG/HFD	Trend	DMY/HFD	Trend
1.	1,3-Dihydroxyacetone	1.87^*∗∗*^	↑	1.07	—	0.88	—
2.	2′-Deoxyadenosine	*N* ^*∗∗*^	*N*	0.44^*∗∗*^	↓	0.42^*∗∗*^	—
3.	3-Hydroxybutyrate	1.72^*∗∗*^	↑	0.89	—	0.80^*∗*^	—
4.	3-Methyl-2-oxovalerate	2.83^*∗*^	↑	1.72^*∗∗*^	↑	1.15^*∗*^	—
5.	4-Aminobutyrate	0.34^*∗∗*^	↓	1.53	↑	1.93	↑
6.	Acetate	0.39^*∗∗*^	↓	1.44^*∗∗*^	↑	1.25	↑
7.	Acetoacetate	0.16^*∗∗*^	↓	0.10^*∗∗*^	↓	0.10^*∗∗*^	—
8.	Acetone	0.33^*∗∗*^	↓	19.63^*∗∗*^	↑	16.18^*∗∗*^	↑
9.	Adenine	2.75^*∗∗*^	↑	53.55^*∗∗*^	↑	38.93^*∗∗*^	↑
10.	Alanine	0.74^*∗∗*^	↓	0.46^*∗∗*^	↓	0.40^*∗∗*^	↓
11.	Allantoin	1.62^*∗∗*^	↑	0.14^*∗∗*^	↓	0.13^*∗∗*^	—
12.	Ascorbate	0.73	↓	0.15^*∗*^	↓	0.07^*∗∗*^	—
13.	Asparagine	1.95^*∗∗*^	↑	1.78^*∗∗*^	↑	1.28^*∗∗*^	↑
14.	Aspartate	1.17	—	2.10^*∗∗*^	↑	1.43^*∗∗*^	↑
15.	Betaine	0.73^*∗∗*^	↓	0.84	—	1.34^*∗*^	↑
16.	Choline	1.82^*∗∗*^	↑	1.56^*∗*^	↑	1.19^*∗*^	↑
17.	Creatine	2.33^*∗∗*^	↑	0.87^*∗*^	—	0.81^*∗*^	—
18.	Cytidine	*N* ^*∗∗*^	*N*	0.19^*∗∗*^	↓	0.14^*∗∗*^	↓
19.	Dimethylamine	0.58^*∗∗*^	—	1.95^*∗∗*^	↑	1.48^*∗∗*^	↑
20.	Ethanol	1.10	—	38.32^*∗∗*^	↑	24.37^*∗∗*^	↑
21.	Ethanolamine	0.61^*∗∗*^	↓	0.19^*∗∗*^	↓	0.15^*∗∗*^	—
22.	Formate	0.20^*∗∗*^	↓	6.27^*∗∗*^	↑	10.62^*∗∗*^	↑
23.	Fumarate	0.15^*∗∗*^	↓	216.28^*∗∗*^	↑	166.51^*∗∗*^	↑
24.	Glucose	0.14^*∗∗*^	↓	0.74^*∗*^	—	0.56^*∗∗*^	—
25.	Glutamate	1.33^*∗∗*^	↑	1.15	—	0.94	—
26.	Glutamine	1.58^*∗∗*^	↑	1.45^*∗∗*^	↑	1.03	—
27.	Glycine	1.34^*∗∗*^	↑	0.68^*∗∗*^	↓	0.46^*∗∗*^	↓
28.	Histidine	0.96	—	0.17^*∗∗*^	↓	0.17^*∗∗*^	↓
29.	Hypoxanthine	0.40^*∗∗*^	↓	0.51^*∗*^	↓	0.73^*∗*^	↓
30.	Inosine	*N* ^*∗∗*^	*N*	62.53^*∗∗*^	↑	38.79^*∗∗*^	↑
31.	Isoleucine	2.13^*∗∗*^	↑	0.01^*∗∗*^	↓	0.01^*∗∗*^	↓
32.	Lactate	0.71^*∗∗*^	↓	0.98	—	0.87	—
33.	Leucine	2.29^*∗∗*^	↑	2.00^*∗∗*^	↑	1.34^*∗*^	↑
34.	Lysine	1.37^*∗∗*^	↑	2.14^*∗∗*^	↑	1.23^*∗*^	↑
35.	Malate	0.22^*∗∗*^	↓	0.12^*∗∗*^	↓	0.85	—
36.	Maltose	0.00^*∗∗*^	↓	0.24^*∗∗*^	↓	0.25^*∗∗*^	↓
37.	Mannose	0.39^*∗∗*^	↓	0.17^*∗∗*^	↓	0.34^*∗∗*^	↓
38.	Methanol	1.32^*∗∗*^	↑	25.28^*∗∗*^	↑	18.04^*∗∗*^	↑
39.	Methionine	2.02^*∗∗*^	↑	0.01^*∗∗*^	↓	0.01^*∗∗*^	↓
40.	*N*,*N*-Dimethylglycine	0.60^*∗*^	↓	17.43^*∗∗*^	↑	16.56^*∗∗*^	↑
41.	Niacinamide	0.42^*∗∗*^	↓	1.61^*∗∗*^	↑	1.61^*∗∗*^	↑
42.	O-Phosphocholine	0.68	↓	8.68^*∗∗*^	—	5.31^*∗∗*^	↑
43.	Ornithine	1.00	—	0.06^*∗∗*^	↓	0.05^*∗∗*^	↓
44.	Pantothenate	0.42^*∗∗*^	↓	46.76^*∗∗*^	↑	30.36^*∗∗*^	↑
45.	Phenylalanine	2.23^*∗∗*^	↑	1.78^*∗*^	↑	1.21	↑
46.	Proline	1.07	—	0.38^*∗∗*^	↓	0.27^*∗∗*^	↓
47.	Pyroglutamate	1.61^*∗*^	↑	1.76^*∗*^	↑	1.93^*∗∗*^	↑
48.	Ribose	0.41^*∗∗*^	↓	0.01^*∗∗*^	↓	0.02^*∗∗*^	↓
49.	Sarcosine	0.58^*∗*^	↓	355.17^*∗∗*^	↑	251.75^*∗∗*^	↑
50.	Serine	1.07	—	0.31^*∗∗*^	↓	0.24^*∗∗*^	↓
51.	Succinate	10.53^*∗∗*^	↑	2.80^*∗∗*^	↑	2.84^*∗∗*^	↑
52.	Taurine	1.92^*∗∗*^	↑	2.56^*∗∗*^	↑	1.72^*∗∗*^	↑
53.	Threonine	1.56^*∗∗*^	↑	0.04^*∗∗*^	↓	0.03^*∗∗*^	↓
54.	Thymidine	*N* ^*∗∗*^	*N*	4.07^*∗∗*^	↑	3.70^*∗∗*^	↑
55.	Thymine	13.60^*∗∗*^	↑	0.03^*∗∗*^	↓	0.02^*∗∗*^	↓
56.	Trimethylamine	0.44^*∗∗*^	↓	788.09^*∗∗*^	↑	498.96^*∗∗*^	↑
57.	Tryptophan	2.37^*∗∗*^	↑	8.43^*∗∗*^	↑	5.74^*∗∗*^	↑
58.	Tyrosine	3.17^*∗∗*^	↑	0.29^*∗∗*^	↓	0.25^*∗∗*^	↓
59.	Uracil	0.69^*∗∗*^	↓	4.24^*∗∗*^	↑	4.22^*∗∗*^	↑
60.	Urea	0.38^*∗∗*^	↓	0.07^*∗∗*^	↓	0.08^*∗∗*^	↓
61.	Uridine	0.16^*∗∗*^	↓	88.88^*∗∗*^	↑	60.22^*∗∗*^	↑
62.	Valine	1.87^*∗∗*^	↑	0.00^*∗∗*^	↓	0.01^*∗∗*^	↓
63.	Xanthosine	0.40^*∗∗*^	↓	16.82^*∗∗*^	↑	14.64^*∗∗*^	↑
64.	Myo-inositol	*N* ^*∗∗*^	*N*	2.03^*∗∗*^	↑	1.40^*∗*^	↑
65.	sn-Glycero-3-phosphocholine	2.01^*∗∗*^	↑	3.06^*∗∗*^	↑	2.09^*∗∗*^	↑
66.	*β*-Alanine	1.32^*∗*^	↑	1.35^*∗*^	↑	0.86	—

Note: ^*∗*^compared with HFD, *p* < 0.05; ^*∗∗*^compared with HFD, *p* < 0.01; *N* means that it cannot be calculated.

## Data Availability

The data used to support the findings of this study are available from the corresponding author upon request.

## Abbreviations

AG: *Ampelopsis grossedentata* (Hand-Mazz) W. T. Wang
DMY: Dihydromyricetin
TCA: Tricarboxylic acid
CoA: Coenzyme A
TMA: Trimethylamine
TMAO: Trimethylamine *N*-oxide.

## Supplementary Materials

Supplementary Materials Figure 1: representative 600 MHz ^1^H-NMR spectra and PLS-DA analysis of ^1^H-NMR spectral data. Figure 1: representative ^1^H-NMR spectra at 600 MHz of the liver (a) and plasma (b) from golden hamster dosed with high-fat diet supplemented with *Ampelopsis grossedentata* group (2 g/kg.d). Figure 2: liver ^1^H NMR spectrum: (a) normal diet (ND); (b) high-fat diet (HFD); (c) high-fat diet supplemented with *Ampelopsis grossedentata* (HFD + AG); (d), high-fat diet supplemented with dihydromyricetin group (HFD + DMY). Table 1: metabolic pathway analysis of golden hamster (HFD vs ND). Table 2: metabolic pathway analysis of golden hamster (HFD vs HFD + AG). Table 3: metabolic pathway analysis of golden hamster (HFD vs HFD + DMY).
