# Correction to “Metabolomics of the Protective Effect of *Ampelopsis grossedentata* and Its Major Active Compound Dihydromyricetin on the Liver of High‐Fat Diet Hamster”

**DOI:** 10.1155/ecam/9801743

**Published:** 2026-02-17

L. Fan. X. Qu, T. Yi, et al., “Metabolomics of the Protective Effect of *Ampelopsis grossedentata* and Its Major Active Compound Dihydromyricetin on the Liver of High‐Fat Diet Hamster,” *Evidence-Based Complementary and Alternative Medicine*, no. 2020 (2020): 1–15. https://doi.org/10.1155/2020/3472578.

In the article, several errors were identified in the notation of chemical structures shown in Figure 3. The corrected Figure 3 is as follows.

**FIGURE 3:**
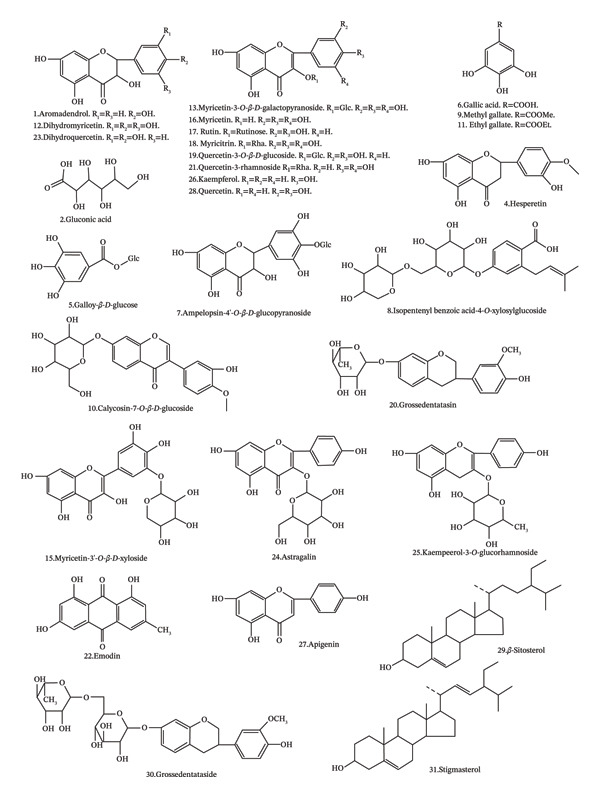
Structures of dihydromyricetin and identified compounds isolated from *Ampelopsis grossedentata*.

**TABLE 1:** List of original edition and corrections of the identified errors in the notation of chemical structures shown in Figure 3.

No.	Original edition	Corrections
1.	No. 6. Apigenin	Changed to No. 27
2.	No. 7. Isopentenylbenzoic acid‐4‐*O*‐xyloosylglucoside	Changed to Ampelopsin‐4′‐O‐β‐D‐glucopyranoside.
3.	No. 8. Gallic acid	Changed to No. 6
4.	No. 11. Methyl gallate	Changed to No. 9
5.	No. 12. Calycosin‐7‐*O*‐β‐*D*‐glucoside	Changed to No. 10
6.	No. 13. Ethyl gallate	Changed to No. 11
7.	No. 14. Dihydromyricetin	Changed to No. 12
8.	No. 15. Grossedentataside	Changed to No. 30
9.	No. 16. Rutin	Changed to No. 17
10.	No. 17. Myricetin‐3‐O‐β‐D‐galactopyranoside	Changed to No. 13
11.	No. 19. Myricetin‐3′‐*O*‐β‐*D*‐xyloside	Changed to No. 15
12.	No. 20. Myricetrin	Changed to No. 18 Myricitrin
13.	No. 21. Quercetin‐3‐O‐β‐D‐glucoside	Changed to No. 19
14.	No. 22. Grossedentatasin	Changed to No. 20
15.	No. 24. Quercetin	Changed to No. 28
16.	No. 25. Emodin	Changed to No. 22
17.	No. 26. Dihydroquercetin	Changed to No. 23
18.	No. 27. Astragalin	Changed to No. 24
19.	No. 28. Kaempeerol‐3‐*O*‐glucorhamnoside	Changed to No. 25
20.	No. 29. Kaempferol	Changed to No. 26
21.	No. 30. Myricetin	Changed to No. 16
22.		Added No. 8. Isopentenylbenzoic acid‐4‐*O*‐xyloosylglucoside
23.		Added No. 21. Quercetin‐3‐rhamnoside
24.		Added No. 29. β‐Sitosterol
25.		Added No. 31. Stigmasterol

We apologize for these errors.

