# Supplementary material for: Metabolomics of the Protective Effect of Ampelopsis grossedentata and Its Major Active Compound Dihydromyricetin on the Liver of High-Fat Diet Hamster
Source: Evid Based Complement Alternat Med. 2020 Jan 28;2020:3472578. doi: 10.1155/2020/3472578 (PMC7007936; doi:10.1155/2020/3472578)
Supplement: Supplementary Materials — Figure 1: representative 600 MHz 1H-NMR spectra and PLS-DA analysis of 1H-NMR spectral data. Figure 1: representative 1H-NMR spectra at 600 MHz of the liver (a) and plasma (b) from golden hamster dosed with high-fat diet supplemented with Ampelopsis grossedentata group (2 g/kg.d). Figure 2: liver 1H NMR spectrum: (a) normal diet (ND); (b) high-fat diet (HFD); (c) high-fat diet supplemented with Ampelopsis grossedentata (HFD + AG); (d), high-fat diet supplemented with dihydromyricetin group (HFD + DMY). Table 1: metabolic pathway analysis of golden hamster (HFD vs ND). Table 2: metabolic pathway analysis of golden hamster (HFD vs HFD + AG). Table 3: metabolic pathway analysis of golden hamster (HFD vs HFD + DMY). [file 3472578.f1.pdf]

Supplementary materials

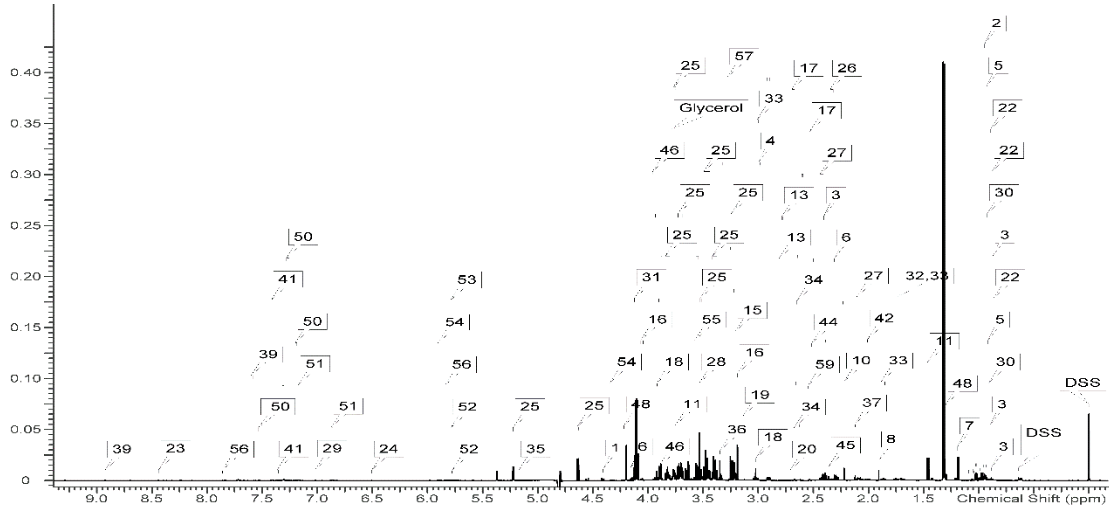

FIGURE 1: Representative 600-MHz 1H-NMR spectra and PLS-DA analysis of 1H-NMR spectral data. FIGURE 1 Representative ^{1}H-NMR spectra at 600 MHz of liver (a) and plasma (b) from golden hamster dosed with high-fat diet supplemented with *Ampelopsis grossedentata* group (2g/kg.d).

1	1,3-Dihydroxyacetone	23	Fumarate	45	Phenylalanine
2	2'-Deoxyadenosine	24	Glucose	46	Proline
3	3-Hydroxybutyrate	25	Glutamate	47	Pyroglutamate
4	3-Methyl-2-oxovalerate	26	Glutamine	48	Ribose
5	4-Aminobutyrate	27	Glycine	49	Sarcosine
6	Acetate	28	Histidine	50	Serine
7	Acetoacetate	29	Hypoxanthine	51	Succinate
8	Acetone	30	Inosine	52	Taurine
9	Adenine	31	Isoleucine	53	Threonine
10	Alanine	32	Lactate	54	Thymidine
11	Allantoin	33	Leucine	55	Thymine
12	Ascorbate	34	Lysine	56	Trimethylamine
13	Asparagine	35	Malate	57	Tryptophan
14	Aspartate	36	Maltose	58	Tyrosine
15	Betaine	37	Mannose	59	Uracil
16	Choline	38	Methanol	60	Urea
17	Creatine	39	Methionine	61	Uridine
18	Cytidine	40	N,N-Dimethylglycine	62	Valine
19	Dimethylamine	41	Niacinamide	63	Xanthosine
20	Ethanol	42	O-Phosphocholine	64	myo-Inositol
21	Ethanolamine	43	Ornithine	65	sn-Glycero-3-phosphocholine
22	Formate	44	Pantothenate	66	β-Alanine

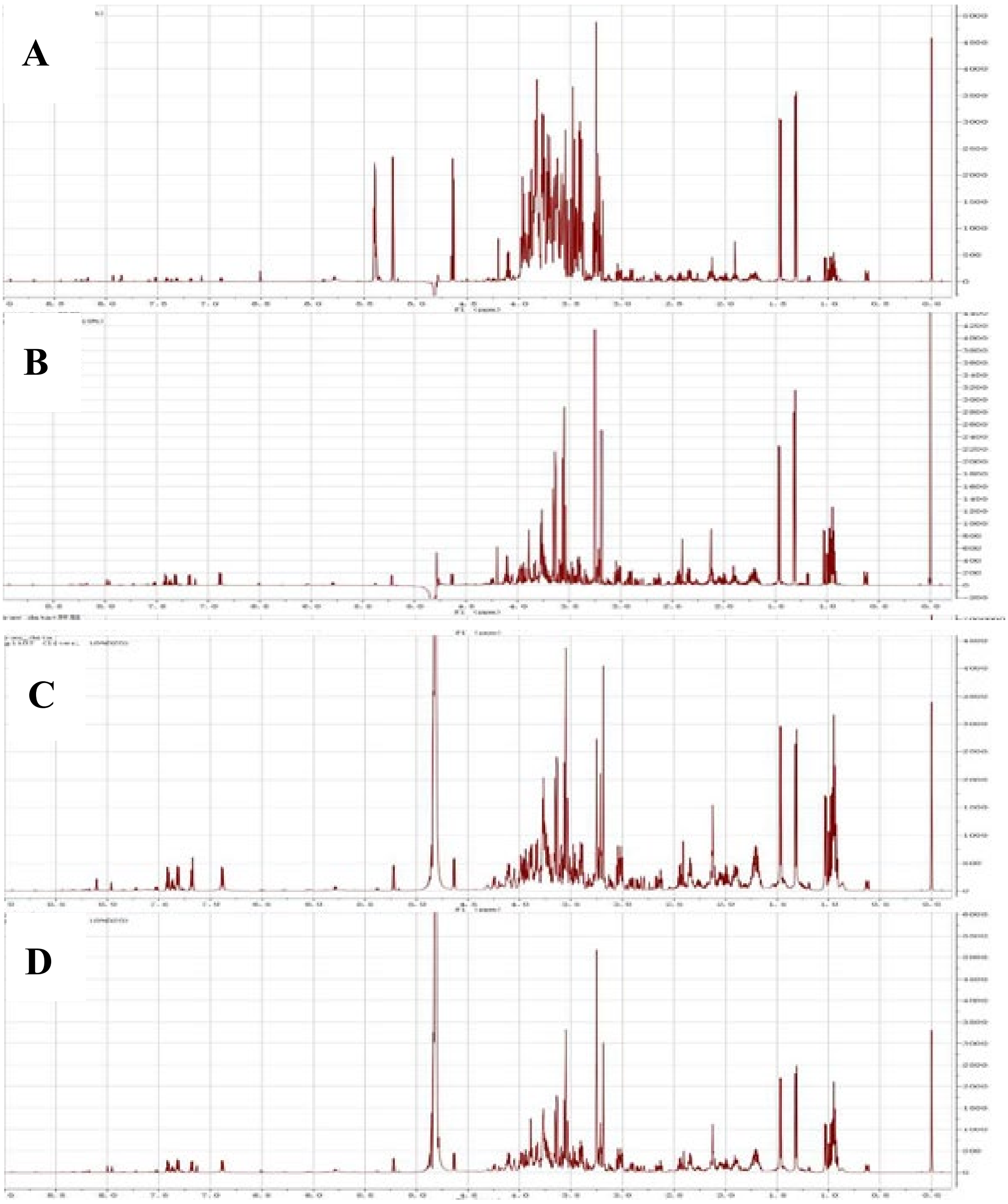

FIGURE 2: Liver ^{1}H NMR spectrum: A, normal diet (ND); B, high-fat diet (HFD); C, high-fat diet supplemented with *Ampelopsis grossedentata* (HFD + AG); D, high-fat diet supplemented with dihydromyricetin group (HFD + DMY).

Table 1: Metabolic Pathway Analysis of Golden Hamster (HFD vs ND).

	Total Cmpd	Hits	Raw p	-log(p)	Holm p	FDR	Impact
Alanine, aspartate and glutamate metabolism	24	8	7E-10	21.08	3E-08	5E-09	0.86
Synthesis and degradation of ketone bodies	6	3	8E-07	14.01	2E-05	2E-06	0.70
Glycine, serine and threonine metabolism	48	10	4E-09	19.25	2E-07	1E-08	0.53
Taurine and hypotaurine metabolism	20	3	2E-07	15.55	5E-06	4E-07	0.36
Arginine and proline metabolism	77	10	7E-08	16.46	2E-06	2E-07	0.36
beta-Alanine metabolism	28	6	2E-05	10.79	3E-04	3E-05	0.26
Pantothenate and CoA biosynthesis	27	5	2E-08	17.52	7E-07	6E-08	0.25
Pyruvate metabolism	32	4	9E-07	13.88	2E-05	2E-06	0.24
Aminoacyl-tRNA biosynthesis	75	18	1E-06	13.50	3E-05	2E-06	0.23
Methane metabolism	34	7	1E-09	20.74	4E-08	5E-09	0.20
Glyoxylate and dicarboxylate metabolism	50	3	3E-10	21.89	2E-08	4E-09	0.17
Lysine degradation	47	2	1E-05	11.20	2E-04	2E-05	0.15
Histidine metabolism	44	3	9E-05	9.35	9E-04	1E-04	0.14
D-Glutamine and D-glutamate metabolism	11	2	1E-05	11.13	2E-04	2E-05	0.14
Pyrimidine metabolism	60	6	4E-12	26.29	2E-10	2E-10	0.13
Glycerophospholipid metabolism	39	4	6E-10	21.21	3E-08	5E-09	0.13
Propanoate metabolism	35	6	3E-09	19.79	1E-07	9E-09	0.13
Phenylalanine metabolism	45	4	5E-10	21.45	2E-08	5E-09	0.12
Ascorbate and aldarate metabolism	45	1	2E-01	1.41	7E-01	3E-01	0.11
Tryptophan metabolism	79	1	3E-06	12.76	6E-05	5E-06	0.11

Table 2: Metabolic Pathway Analysis of Golden Hamster (HFD vs HFD + AG).

	Total Cmpd	Hits	Raw p	-log(p)	Holm p	FDR	Impact
Synthesis and degradation of ketone bodies	6	3	1.52E-10	22.608	3.52E-09	2.60E-10	0.70
Alanine, aspartate and glutamate metabolism	24	7	2.52E-12	26.706	8.32E-11	6.37E-12	0.68
Glycine, serine and threonine metabolism	48	10	3.37E-15	33.323	1.59E-13	2.55E-14	0.53
Taurine and hypotaurine metabolism	20	3	4.21E-09	19.287	5.89E-08	5.57E-09	0.36
D-Glutamine and D-glutamate metabolism	11	2	0.001046	6.8631	0.004183	0.001108	0.35
Arginine and proline metabolism	77	9	4.33E-15	33.074	1.99E-13	2.87E-14	0.32
beta-Alanine metabolism	28	6	1.31E-12	27.364	4.83E-11	4.07E-12	0.26
Pantothenate and CoA biosynthesis	27	5	1.69E-17	38.622	8.43E-16	2.23E-16	0.25
Pyruvate metabolism	32	3	1.78E-06	13.238	1.78E-05	2.15E-06	0.24
Pyrimidine metabolism	60	8	2.75E-10	22.014	5.78E-09	4.42E-10	0.22
Methane metabolism	34	7	1.51E-14	31.821	6.66E-13	8.03E-14	0.20
Aminoacyl-tRNA biosynthesis	75	17	1.39E-17	38.814	7.10E-16	2.23E-16	0.17
Lysine degradation	47	2	1.46E-09	20.343	2.21E-08	1.99E-09	0.15
Glyoxylate and dicarboxylate metabolism	50	2	2.37E-06	12.955	2.13E-05	2.79E-06	0.14
Histidine metabolism	44	2	1.95E-07	15.448	2.15E-06	2.41E-07	0.14
Inositol phosphate metabolism	39	1	0.000174	8.6589	0.001041	0.000192	0.14
Glycerophospholipid metabolism	39	4	3.22E-10	21.858	6.43E-09	5.01E-10	0.13
Propanoate metabolism	35	6	1.80E-18	40.859	9.54E-17	9.54E-17	0.13
Phenylalanine metabolism	45	4	1.51E-12	27.216	5.30E-11	4.22E-12	0.12
Ascorbate and aldarate metabolism	45	2	0.001801	6.3194	0.005403	0.001872	0.11
Tryptophan metabolism	79	1	3.58E-10	21.751	6.80E-09	5.42E-10	0.11

Table 3: Metabolic Pathway Analysis of Golden Hamster (HFD vs HFD + DMY).

	Total Cmpd	Hits	Raw p	-log(p)	Holm p	FDR	Impact
Synthesis and degradation of ketone bodies	6	3	1.90E-12	26.987	7.23E-11	6.31E-12	0.70
Alanine, aspartate and glutamate metabolism	24	7	1.08E-12	27.554	4.65E-11	4.96E-12	0.68
Glycine, serine and threonine metabolism	48	10	1.97E-14	31.558	9.85E-13	2.49E-13	0.53
Taurine and hypotaurine metabolism	20	3	2.00E-07	15.427	3.39E-06	2.86E-07	0.36
D-Glutamine and D-glutamate metabolism	11	2	0.02176	3.8277	0.08704	0.023066	0.35
Arginine and proline metabolism	77	9	2.35E-14	31.383	1.15E-12	2.49E-13	0.32
beta-Alanine metabolism	28	6	1.72E-13	29.39	7.75E-12	1.01E-12	0.26
Pantothenate and CoA biosynthesis	27	5	4.59E-07	14.595	5.96E-06	5.93E-07	0.25
Pyruvate metabolism	32	3	2.47E-07	15.215	3.95E-06	3.44E-07	0.24
Pyrimidine metabolism	60	8	2.67E-11	24.345	8.82E-10	6.75E-11	0.22
Methane metabolism	34	7	3.99E-15	33.154	2.08E-13	1.06E-13	0.20
Aminoacyl-tRNA biosynthesis	75	17	5.57E-11	23.611	1.73E-09	1.28E-10	0.17
Lysine degradation	47	2	1.24E-10	22.814	3.71E-09	2.67E-10	0.15
Glyoxylate and dicarboxylate metabolism	50	2	5.66E-08	16.687	1.19E-06	9.09E-08	0.14
Histidine metabolism	44	2	1.84E-10	22.417	5.15E-09	3.75E-10	0.14
Inositol phosphate metabolism	39	1	0.010427	4.5633	0.052136	0.011278	0.14
Glycerophospholipid metabolism	39	4	1.75E-11	24.767	5.96E-10	4.65E-11	0.13
Propanoate metabolism	35	6	4.25E-07	14.671	5.95E-06	5.63E-07	0.13
Phenylalanine metabolism	45	4	1.12E-12	27.516	4.71E-11	4.96E-12	0.12
Ascorbate and aldarate metabolism	45	2	0.001	6.9075	0.007002	0.001128	0.11
Tryptophan metabolism	79	1	4.38E-10	21.55	1.14E-08	8.28E-10	0.11
